# Renal abnormalities and its associated factors among school-aged children living in *Schistosoma mansoni* endemic communities in Northwestern Tanzania

**DOI:** 10.1186/s41182-020-00243-6

**Published:** 2020-07-06

**Authors:** Neema M. Kayange, Nicholaus Mazuguni, Adolfine Hokororo, Charles Muiruri, Karl Reis, Benson R. Kidenya, Humphrey D. Mazigo

**Affiliations:** 1 Department of Pediatrics, Weill Bugando School of Medicine, Catholic University of Health and Allied Sciences, Mwanza, United Republic of Tanzania; 2 Kilimanjaro Fertility Institute (KFi), Kilimanjaro, Tanzania; 3 Department of Biochemistry and Molecular Biology, Weill Bugando School of Medicine, Catholic University of Health and Allied Sciences, Mwanza, United Republic of Tanzania; 4 Department of Medical Parasitology and Entomology, Weill Bugando School of Medicine, Catholic University of Health and Allied Sciences, Mwanza, United Republic of Tanzania; 5 Department of Population Health Sciences, Duke University, Durham, NC USA; 6 Centre for Global Health at Weill Cornell, New York, USA; 7 Department of Epidemiology and Biostatistics, Kilimanjaro Christian Medical University College, Moshi, Tanzania

**Keywords:** Renal abnormalities, Schistosomiasis, *Schistosoma mansoni*, School-aged children, Tanzania

## Abstract

**Background:**

In sub-Saharan Africa, renal abnormalities are a major public health concern, especially in children living in *Schistosoma haematobium* endemic areas. However, there is a dearth of data on renal abnormalities among children living in *Schistosoma mansoni* endemic areas. The objective of the study was to assess the prevalence of renal abnormalities among school children in a *Schistosoma mansoni* endemic community in Northwestern Tanzania.

**Methods:**

A cross-sectional study was conducted between January and March 2017 among school children aged 6–13 years, attending three primary schools located along the shoreline of Lake Victoria. A single urine sample was collected from each child and screened for *S. mansoni* using circulating cathodic antigen and for *S. haematobium* eggs using a urine filtration technique. A urine dipstick was used to screen for urine protein levels, creatinine levels, microalbuminuria, and red blood cells. Venous blood was obtained for estimation of creatinine level and for malaria diagnosis. The primary outcomes were the prevalence of renal abnormalities, defined by the presence of low estimated glomerular filtration rate (eGFR), proteinuria or microalbuminuria, and hematuria in urine.

**Results:**

Of 507 children included in the final analysis, 49.9% (253/507) were male with a mean age of 8.51 ± 1.3 years. Overall, 64.0% (326/507) of the children were infected with *S. mansoni*, and 1.6% (8/507) of the children were infected with *S. haematobium*. A total of 71 (14%) of the children had proteinuria, 37 (7.3%) had hematuria, and 8 (1.6%) had a low estimated glomerular filtration rate (eGFR). Overall prevalence of renal abnormalities was 22.9%. Renal abnormalities (proteinuria) were associated with *S. mansoni* infection (OR = 4.9, 95% CI 2.1–11.2, *p* < 0.001) and having red blood cells in urine (OR = 5.3, 95% CI 2.5–11.2, *p* < 0.001).

**Conclusion:**

Twenty-two percent of school children who participated in this study had renal abnormalities associated with *S. mansoni* infection. Given the high prevalence of *S. mansoni*, longitudinal epidemiological surveillance is warranted to measure the burden of renal abnormalities and assess the impact of the praziquantel treatment on these abnormalities.

## Background

Early detection and identification of renal diseases in children and adolescents is very important in the prevention of chronic renal diseases. Childhood renal diseases can lead to treatable disorders without long-term consequences and in other cases life-threatening conditions for children [1]. Proteinuria/microalbuminuria and hematuria are important early markers for progression to end-stage renal disease and cardiovascular diseases [2, 3].

In Africa, the causes of renal diseases in children are multifactorial [4]. These causes range from non-infectious diseases (intrauterine injury to the kidney, malnutrition, and sickle cell nephropathy) to infectious diseases (malaria, post-infection glomerulo-nephritis, and HIV nephropathy) [5, 6]. Studies from sub-Saharan Africa have reported the association of *S. haematobium* with renal abnormalities. The presence of hematuria has remained an important marker of renal disease associated with *S. haematobium* [7]. However, the association between *S. mansoni* and renal abnormalities is poorly understood. A few studies have reported the associations between both *S. haematobium* and *S. Mansoni* and markers of renal abnormalities such as hematuria and proteinuria [8, 9]. When diagnostic tests were repeated in the same location, years after treatment for *S. haematobium*, a lower prevalence of proteinuria and hematuria was consistently observed [10, 11]. Kayange et al. reported that *S. mansoni* was highly associated with proteinuria in a hospital-based study [12]. The mechanisms behind the association of *S. mansoni* and renal abnormalities can be explained by deposition of immune complex formed by *Schistosoma* antigen and IgG/IgM antibodies in the glomerular basement membrane [13, 14].

In the Mwanza region, previous studies have demonstrated that there is a high prevalence of schistosomiasis, which contributes to a high prevalence of persistence proteinuria and later to a chronic kidney disease [12]. Therefore, the objective of the study was to assess the prevalence of renal abnormalities based on creatinine, proteinuria, and hematuria levels in children living in an endemic *S. mansoni* community.

## Methods

### Study area

The study was conducted at Ilemela district of Mwanza region in Northwestern Tanzania. The region has 139 primary schools which enroll over 95% of all school-aged children in the region [15]. Specifically, the study was conducted in Ilemela District at Kayenze, Kabangaja, and Sangabuye primary schools located in Kayenze, Sangabuye, and Bugongwa villages respectively. These schools were selected because they are located close to the shores of Lake Victoria where previous studies have reported a high prevalence of intestinal schistosomiasis [16]. Communities in this area are at an increased risk of schistosomiasis infection because of daily activities such as bathing/swimming, washing cloth, and fetching water for domestic use from the lake [17]. Primary school children in this area receive an annual mass drug administration of praziquantel to control schistosomiasis infection.

### Study design, population, and inclusion and exclusion criteria

We conducted a cross-sectional study among school children between January and March 2017 at the three primary schools. Standard II class (second grade) pupils were enrolled in the study because for this class, no praziquantel had been administered in the previous year. We excluded children with fever since fever is known to cause proteinuria. We also excluded children with preexisting renal disease since it is difficult to determine acute kidney injury in a child with preexisting renal disease without serial creatinine measurement.

### Sample size calculation

Our sample size was 507. We calculated the sample size using the Yamane Taro formula (1967) $n=\frac{N}{1+N{e}^2}$, where *n* is the sample size, *N* is the population size of all standard II pupils in the district (94,000), and *e* is the level of precision at a 95% confidence level, and *p* = 0.05 is assumed for the equation [18].

### Sampling technique

Three villages alongside Lake Victoria and their corresponding primary schools were selected based on convenience and feasibility. A systematic sampling method was used to select study participants, using the class register as a sampling frame. Attempt was made to sample an equal number of girls and boys by reviewing participant’s recruitment logs.

### Data collection

A week before urine and blood sample collection, the study objectives were explained to the teachers and children. The children were then provided with informed consent forms to take home to their parents/guardians. They were instructed to tell their parents/guardians to read the informed consent forms and sign if they had understood and agreed to their child’s participation. The signed forms were then brought back to school, and the meeting was also held between the study team and the children and their parents/guardians to facilitate understanding of the objective of the study and associated risks and benefits of participation.

### Physical examination of school children

A brief physical examination was done to check for facial or leg edema, temperature, weight (by digital weighing machine), and height. Blood pressure was measured in an enclosed room using a pediatric digital blood pressure monitor which was calibrated before each use. Hypertension was determined using the World Health Organization age-based blood pressure reference charts [19]. The nutritional status of each child was calculated using the WHO 2013 BMI percentile charts according to the child’s age and sex [20].

### Laboratory procedures

#### Urine sampling

A single early morning urine sample was collected from each participating child. Urine albumin and creatinine concentrations were measured using a 25 Biosystems Clinical Chemistry Analyzer according to standard laboratory procedure at the National Institute for Medical Research laboratory, Mwanza, Tanzania. Urine albumin-creatinine ratio (ACR) was calculated and classified using the following categories [21]: normal ≤ 30 mg/g, moderately increased = 30–300 mg/g, and severely increased ≥ 300 mg/g.

#### Proteinuria measurement

Proteinuria was measured using urine dipsticks (Multistix™, Bayer, Germany), and proteinuria level was reported as negative, 1+ (30 mg/dL), 2+ (100 mg/dL), 3+ (300 mg/dL), or 4+ (1000 mg/dL) as per manufacturer instructions. Children were considered to have proteinuria if they scored 2+, 3+, or 4+. Other indicators recorded using a urine dipstick were leukocytes esterase, hematuria, nitrates, glucose, and ketones. The urinalysis was considered positive if any of these indicators were detected in urine samples. Positive nitrate and leukocyte esterase were considered an indicator for urinary tract infection [22].

#### Examination of creatinine level in serum

Serum creatinine level was measured using a Cobas 400 clinical chemistry machine (Roche, Germany), calibrated by the Creatinine Jaffe 2 method. Two milliliters of blood was poured into the test tube and then into the machine. An estimated glomerular filtration rate (eGFR) was calculated using modified Schwartz equation (taking into account estimation of GFR in children using serum creatinine and height) as recommended by the Kidney Disease Improving Global Outcomes (KDIGO) guidelines and validated in children with and without CKD [21, 23, 24].

#### Examination of *Schistosoma mansoni* and *Schistosoma haematobium*

To screen for S*. mansoni* infection, point-of-care circulating cathodic antigen tests (CCA) (rapid medical diagnostic batch number: 170331037) were used. The same urine samples obtained earlier were used for the CCA test [25, 26]. Results were recorded as per manufacturer instructions [26].

For screening of *S. haematobium* infection, a urine filtration technique was used in which the urine sample was filtered (pore aperture 20 μm; Sefar AG, Heiden, Switzerland) and the filter was then placed on a slide and examined under a microscope for presence of *S. haematobium* eggs [27].

#### Malaria diagnosis

Malaria diagnosis was determined using an mRDT (Malaria Antigen P.f/Pan 05FK60, Standard Diagnostics (SD) Bioline, India) and interpreted according to the manufacturer’s instructions.

##### Definition of renal abnormalities

In this study, renal abnormalities were operationally defined as eGFR < 60 ml/min/1.73 m^2^ or microalbuminuria (urine for ACR > 30 mg/g or proteinuria of ≥ 2+ or hematuria ≥ 2+).

### Data analysis

Data were double entered into Microsoft Excel and analyzed using STATA version 15. Results were summarized using proportions (%) for categorical data and means (SD) or medians (IQR) for continuous variables. Categorical variables were compared using either Pearson’s chi-squared or Fisher’s exact test. Determination of predictors of proteinuria and microalbuminuria was done by univariate logistic regressions followed by multivariable logistic regression. Odds ratios (OR) with 95% confidence interval (CI) were reported.

## Results

### Characteristics of the study participants

**Fig. 1:**
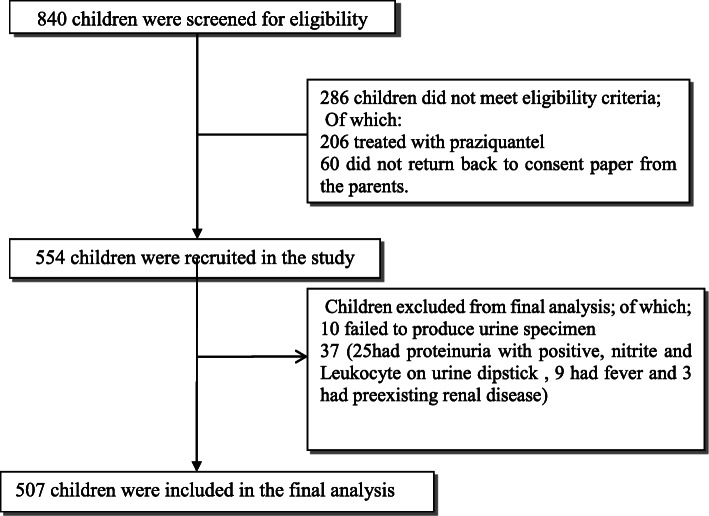
Study enrollment overview

A total of 554 school children from Kayenze, Kabangaja, and Sangabuye primary school were enrolled into the study (Fig. 1). We excluded 47 children because of the following reasons: 25 had features suggestive of UTI (proteinuria, nitrate, and leucocytes on urine dipstick), 10 failed to produce urine specimen, 9 with fever, and 3 children with preexisting renal disease. Five hundred and seven children were included in the final analysis. Of these children, 49.9% (253/507) were male with a mean age of 8.5 ± 1.3 years. The majority of children (84.2%) had been in contact with the lake within 1 week prior to the survey administration, and 60% used the lake as a source of water for bathing, cooking, washing, and drinking (Table 1).

**Table 1:** Characteristics of 507 primary school children

Characteristics	Number	Percentage/median (IQR)
Age (years)	507	8 (8–9)
Female	254	50.1
Type of water source		
Lake or pond water	304	60.0
Tap water	203	40.0
Contact with lake in the past 1 week		
Yes	427	84.2
No	80	15.8
Ethnicity		
African	506	99.8
Asian	1	0.2

### Prevalence of schistosomiasis and malaria

The prevalence of *S. mansoni* and *S. haematobium* was 64.3% and 1.6% respectively*.* Prevalence of *S. mansoni* was higher in younger children 6 to 9 years compared to participants 10 to 13 years (76% vs. 23%, *p* = 0.062), but the prevalence did not differ by sex (50% vs. 49%, *p* = 0.538). The prevalence of asymptomatic malaria was 17.4% (88/507). Males were slightly more affected than females (56% and 43%). Younger children aged 6 to 9 years were also more affected by malaria than older children aged 10 to 13 years although this was not statistically significant (*p* = 0.153).

### Renal abnormalities

Eight (1.6%) of the study participants had an eGFR less than 60, which meets the criteria for severe renal abnormalities. Twenty (3.9%) participants had an eGFR of < 90 ml/min/1.73 m^3^. Due to the small number of cases with eGFR < 60, this study could not conduct univariate and multivariate analysis. Overall, 33 (6.5%) participants had microalbuminuria in the nephrotic range with severely increased ACR (> 300gm/g), and 241 (47.5%) had moderately increased ACR range 30–300 mg/g. Seventy-one (71/507) (14.0%) participants had proteinuria of 2+ to 4+, and 37 (7.3%) had blood in their urine. Prevalence of renal abnormalities was 22.9% (Table 2).

**Table 2:** Laboratory renal abnormalities among 507 study participants

Variable	Number	Percent (%)
eGFR (mL/min/1.73 m^2^)		
> 90	487	96.1
60–89	12	2.4
30–59	6	1.2
15–29	2	0.4
Proteinuria		
Protein negative	271	53.5
Protein +1 (trace)	165	32.5
Protein +2	22	4.3
Protein +3	40	7.9
Protein +4	9	1.8
Blood in urine		
Negative	467	92.1
+1	3	0.6
+2	25	4.9
+3	12	2.4
Renal dysfunction	116	22.9
ACR		
Normal to mild increased	233	46.0
Moderately increased	241	47.5
Severely increased	33	6.5

### Factors associated with proteinuria

All presenting demographic and clinical characteristics were evaluated as possible factors predictive of proteinuria. Significant predictors of proteinuria on multivariable analysis included schistosomiasis and blood in the urine. We observed that children infected with *S. mansoni* had 4 times higher odds of having proteinuria compared to those without *S. mansoni* (OR [95% CI] = 4.9 [2.1–11.2], *p* < 0.001). Also, children with blood in their urine had five times higher odds of having proteinuria compared with those without blood in their urine (OR [95% CI] = 5.3 [2.5–11.0], *p* < 0.001 (Table 3).

**Table 3:** Factors associated with proteinuria

Variable	Yes (*n*, %)	No (*n*, %)	Univariate, OR [95% CI]	*p* value	Multivariate, OR [95% CI]	*p* value
Age						
6–9 years	55 (13.7)	348 (86.4)	1.0			
10–13 years	16 (15.4)	88 (84.6)	1.2 [0.6–2.1]	0.649	0.8 [0.4–1.6]	0.520
Sex						
Female	29 (11.4)	225 (88.6)	1.0			
Male	42 (16.6)	211 (83.4)	1.5 [0.9–2.6]	0.094	1.5 [0.9–2.5]	0.129
Water source						
Tap	26 (12.8)	177 (87.2)	1.0			
Lake water	45 (14.8)	259 (85.2)	1.2 [0.7–2.0]	0.526	1.1 [0.6–2.1]	0.694
Contact to lake within 1 week						
No	11 (14.0)	69 (86.0)	1.0			
Yes	60 (14.0)	367 (86.0)	1.0 [0.5–2.0]	0.943	0.9 [0.4–2.1]	0.831
Schistosomiasis by cca						
No	7 (3.9)	174 (96.1)	1.0			
Yes	64 (19.6)	262 (80.4)	6.1 [2.7–13.6]	< 0.001	4.9 [2.1–11.2]	< 0.001
RBCs in urine						
No RBCs	53 (11.3)	417 (88.7)	1.0			
RBCs in urine	18 (48.6)	19 (51.4)	7.4 [3.7–15.1]	< 0.001	5.3 [2.5–11.2]	< 0.001
Malaria						
Negative	63 (15.0)	356 (85.0)	1.0			
Positive	8 (9.9)	80 (90.1)	0.6 [0.3–1.2]	0.149	0.8 [0.3–1.7]	0.528
Nutrition status						
Normal	55 (13.5)	352 (86.5)	1.0			
Moderate malnutrition	10 (15.2)	56 (84.9)	1.1 [0.6–2.4]	0.720	1.0 [0.4–2.1]	0.967
Severe malnutrition	6 (17.7)	28 (82.3)	1.4 [0.5–3.5]	0.504	1.0 [0.3–2.8]	0.992
Haematobium						
Negative	68 (14.0)	431 (86.0)	1.0			
Positive	3 (37.5)	5 (86.0)	3.8 [0.9–16.3]	0.072	3.5 [0.6–19.8]	0.173

### Factors associated with microalbuminuria

Presence of blood in urine was the only factor associated with microalbuminuria on multivariable analysis in this study. Children with blood in the urine had two times higher odds of having microalbuminuria compared to those without blood in their urine (OR [95% CI] 2.3 [1.1–4.9], *p* = 0.035 (Table 4).

**Table 4:** Factors associated with microalbuminuria

	Microalbuminuria		Univariate OR [95% CI]	*p* value	Multivariate OR [95% CI]	*p* value
Variable	Yes (*n* %)	No (*n* %)				
Age						
6–9 year	215 (53.3)	188 (46.7)	1.0			
10–13 year	59 (56.7)	45 (43.3)	1.0 [0.9–1.2]	0.470	1.1 [0.6–1.6]	0.820
Sex						
Female	140 (55.1)	114 (44.9)	1.0			
Male	134 (53.0)	119 (47.0)	0.9 [0.6–1.3]	0.627	0.8 [0.6–1.2]	0.357
Water source						
Lake water	115 (56.7)	88 (43.3)	1.0			
Tap	159 (52.4)	145 (47.7)	0.8 [0.6–1.2]	0.336	0.9 [0.6–1.4]	0.762
Contact to lake within 1 week						
No	49 (61.2)	31 (38.8)	1.0			
Yes	202 (47.3)	225 (52.7)	0.7 [0.4–1.1]	0.160	0.7 [0.4–1.2]	0.205
Schistosomiasis by cca						
No	93 (51.4)	88 (48.6)	1.0			
Yes	181 (55.5)	145 (44.5)	1.2 [0.8–1.7]	0.370	1.2 [0.8–1.7]	0.442
RBCs in urine						
No RBCs	247 (52.6)	223 (447.4)	1.0			
RBCs in urine	27 (73.0)	10 (27.0)	2.4 [1.2–5.2]	0.020	2.3 [1.1–4.9]	0.035
Malaria						
Negative	219 (52.3)	200 (47.7)	1.0			
Positive	55 (62.5)	33 (37.5)	1.5 [1.0–2.4]	0.081	1.8 [1.0–2.9]	0.024
Nutrition status						
Normal	216 (53.1)	191 (46.9)				
Moderate malnutrition	38 (57.6)	28 (42.4)	1.2 [0.7–2.0]	0.496	1.3 [0.7–2.1]	0.414
Severe malnutrition	20 (58.8)	14 (41.2)	1.3 [0.6–2.6]	0.519	1.2 [0.6–2.6]	0.573
Haematobium						
Negative	268 (53.7)	231 (43.3)	1.0			
Positive	6 (75.0)	2 (25.0)	2.6 [0.5–12.9]	0.246	2.5 [0.5–12.9]	0.278

## Discussion

In this study, the overall prevalence of renal abnormalities was 22.9% and factors associated with proteinuria were *S. mansoni* infection and RBCs in urine.

Based on CCA, close to two thirds of the children participating in this study were infected with *S. mansoni* with a small percentage infected with *S. haematobium*. Similarly, a marginal proportion of children were diagnosed with proteinuria and hematuria, all of whom had low eGFR.

Based on eGFR, only a small percentage of the children included in the present study had renal abnormalities. The observed prevalence was lower than the 7.4% reported in another study done in Northwestern Tanzania and 4.6 reported in a systematic study done in Africa [12, 28]. This variation could be due to the different age group or type of exposure of participants in this study.

Although urogenital schistosomiasis is known to contribute to renal abnormalities, no association was observed between *S. haematobium* and low eGFR in this study. This can partly be explained by the low prevalence of *S. haematobium* found, as studies in areas with a higher prevalence of *S. haematobium* have reported an association between low eGFR and *S. haematobium* [7].

On the other hand, the prevalence of proteinuria observed in the present study was lower than what was observed in previous studies which range from 32 to 44% in the same region [7, 29]. This may be due to the fact that previous studies involved children with sickle cell anemia and HIV infection and our study was done in the general population of children in primary schools.

Microalbuminuria is known to be an early predictive factor for renal and cardiovascular diseases, not only for patients with diabetes mellitus, hypertension, or sickle cell anemia, but also in the general population [30]. In this study, almost half of the participants had moderately increased ACR and 6.5% had severely increased ACR. This was much higher than the levels that were found among Korean children aged 5–14 years [31]. It is unclear why there is such difference, and future research should investigate reasons for disparity.

Hematuria found in 37 participants may have originated from the glomerular, renal tubules and interstitial space, or urinary tract. In children with *S. haematobium*, the deposition of eggs in the bladder and ureter and subsequent granulomatous inflammation cause hematuria [32, 33]. Studies in Africa have reported rates of hematuria ranging from 0.6 to 67%, though most of the studies were done more than 20 years ago in areas with high prevalence of *S. haematobium* [7].

Our study showed an alarmingly high *S. mansoni* prevalence of 64% among primary school children living along the shores of Lake Victoria. We used a more sensitive test point-of-care CCA test, which is reported to be more sensitive than Kato Katz slides (86% sensitive versus 62%) [25]. Similar prevalence of 62% and 64% was observed in Sengerema and Ukerewe, two nearby communities, using a mixed method of POC-CCA and Kato Katz slides [34]. The high prevalence of schistosomiasis found here can be explained by an inadequate clean water supply, poor sanitation, domestic activities such as farming and fishing, recreational swimming, and also a high density of intermediate hosts along Lake Victoria [17].

In the present study, the prevalence of *S. haematobium* by single filtration test was low. This prevalence is slightly lower compared to what was reported in Zanzibar (Unguja and Pemba), in which the prevalence of 2.7% and 7% in primary school children was found [35]. The low prevalence may be because the location of communities in our study was along the lakeshores, which are less affected by *S. haematobium* [17]. The different species of snail hosts that transmit *Schistosoma* species have different preferences for location, with those transmitting *S. mansoni* preferring larger bodies and those transmitted *S. haematobium* preferring small water bodies away from the lakeshore line [17].

*S. mansoni* infection was strongly associated with proteinuria. Participants with proteinuria had four times higher odds of having proteinuria compared to those with no infection. Glomerular lesion associated with *S. mansoni* results from circulating antigen of *Schistosoma* eggs. The immune complex when deposited in the glomerulus results in injury and a cascade of immunological reaction. Previous studies in sub-Saharan Africa have reported similar findings [7].

Our studies had several limitations. First, renal ultrasound could have been helpful in identifying morphological changes consistent with schistosomiasis but was not done in this study. Second, this was a cross-sectional study with no control group; therefore, causality in the relationship between kidney injury and schistosomiasis infection could not be examined. Third, children with fever and preexisting renal disease were excluded; this may limit the generalization of our results. Also, identification of schistosomiasis using a gold standard test (stool microscopy) was not done in this study. Despite these limitations, this study contributed to the few studies that have evaluated the relationship between schistosomiasis and renal abnormalities.

## Conclusion

Our study findings show that 1 out 5 of primary school children have renal abnormalities. The study also identified a very high prevalence of *S. mansoni* and low prevalence of *S. haematobium*. Children with schistosomiasis had higher odds of having proteinuria. Screening and mass treatment of schistosomiasis should be a priority in this community in order to prevent long-term effect of renal injury. We also recommend longitudinal studies to identify causal relationship between *S. mansoni* and renal abnormalities.

## Data Availability

The datasets collected and/or analyzed during the current study are available from the corresponding author upon request.

## Abbreviations

ACR: Urine for albumin creatinine ration
mRDT: Malaria rapid diagnostic test
eGFR: Glomerular filtration rate
CI: Confidence interval
IQR: Interquartile range

## References

[1] Ardissino G, Dacco V, Testa S, Bonaudo R, Claris-Appiani A, Taioli E, Marra G, Edefonti A, Sereni F, ItalKid P (2003). Epidemiology of chronic renal failure in children: data from the ItalKid project. Pediatrics.

[2] Hemmelgarn BR, Manns BJ, Lloyd A, James MT, Klarenbach S, Quinn RR, Wiebe N, Tonelli M (2010). Alberta Kidney Disease Network.: Relation between kidney function, proteinuria, and adverse outcomes. JAMA.

[3] Hillege HL, Fidler V, Diercks GF, van Gilst WH, de Zeeuw D, van Veldhuisen DJ, Gans RO, Janssen WM, Grobbee DE, de Jong PE (2002). Urinary albumin excretion predicts cardiovascular and noncardiovascular mortality in general population. Circulation.

[4] Ali el-TM., Abdelraheem MB., Mohamed RM., Hassan EG., Watson AR: Chronic renal failure in Sudanese children: aetiology and outcomes. Pediatric Nephlorology 2009, 24(2):349-353. 10.1007/s00467-008-1022-8 18958501

[5] Zohdi V, Sutherland MR, Lim K, Gubhaju L, Zimanyi MA, Black MJ. Low birth weight due to intrauterine growth restriction and/or preterm birth: effects on nephron number and long-term renal health. Int J Nephrol. 2012;136942. 10.1155/2012/136942 PMC3434386 22970368

[6] Asinobi AO, Ademola AD, Ogunkunle OO, Mott SA (2014). Paediatric end-stage renal disease in a tertiary hospital in South West Nigeria. BMC Nephrol.

[7] Kayange NM, Smart LR, Tallman JE, Chu EY, Fitzgerald DW, Pain KJ, Peck RN (2015). Kidney disease among children in sub-Saharan Africa: systematic review. Pediatr Res.

[8] Johansen MV, Simonsen PE, Butterworth AE, Ouma JH, Mbugua GG, Sturrock RF, Orinda DA, Christensen NO (1994). A survey of Schistosoma mansoni induced kidney disease in children in an endemic area of Machakos District. Kenya. Acta Trop.

[9] Adesola A, Akibu O, Ademola O, Akinwale A (2008). Haematuria in the rural primary school children in South Western Nigeria using Combi test strips. Res J Med Sci.

[10] Heurtier Y, Lamothe F, Develoux M, Docquier J, Mouchet F, Sellin E, Sellin B (1986). Urinary tract lesions due to Schistosoma haematobium infection assessed by ultrasonography in a community based study in Niger. Am J Trop Med Hyg.

[11] King CH, Muchiri EM, Ouma JH (1992). Age-targeted chemotherapy for control of urinary schistosomiasis in endemic populations. Mem Inst Oswaldo Cruz.

[12] Kayange NM, Smart LR, Downs JA, Maskini M, Fitzgerald DW, Peck RN (2015). The influence of HIV and schistosomiasis on renal function: a cross-sectional study among children at a hospital in Tanzania. PLoS Negl Trop Dis.

[13] Geraldo Bezerra da Silva Junior, Ana Amélia Reis Jereissati, Ane Karoline Medina Neri, Danielli Oliveira da Costa Lino, Juliana Gomes Ramalho de Oliveira, Elizabeth De Francesco Daher.: Neglected tropical diseases with an impact on kidney function. , Current Topics in Tropical Emerging Diseases and Travel Medicine, Alfonso J Rodriguez-Morales, IntechOpen, 2018.

[14] Da Silva GB (2013). Junior., Duarte DB., Barros EJG., Daher EDF.: Schistosomiasis-associated kidney disease: a review. Asian Pac J Trop Dis.

[15] Tanzania National Bureau of Statistics: Tanzania populations census 2012Tanzania Government 2012.

[16] Olsen A, Kinung’hi S, Magnussen P (2015). Schistosoma mansoni infection along the coast of Lake Victoria in Mwanza region Tanzania. Am J Trop Med Hyg.

[17] Mazigo HD, Nuwaha F, Kinung’hi SM, Morona D, Pinot de Moira A, Wilson S, Heukelbach J, Dunne DW (2012). Epidemiology and control of human schistosomiasis in Tanzania. Parasit Vectors.

[18] Yamane T.: Statistics: an introductory analysis, 2nd edition, . New York: Harper and Row 1967.

[19] Banker A, Bell C, Gupta-Malhotra M, Samuels J (2016). Blood pressure percentile charts to identify high or low blood pressure in children. BMC Pediatr.

[20] WHO child growth standard (n.d) Child growth standards: BMI-for-age. Available from :http:/www.who.int/childgrowth/standards/bmi-for-age/en/.

[21] Kidney disease:Improving Global Outcomes (KDIGO) CKD Work group (2013) KDIGO 2012 Clinical practice guidelines for the evaluation and management of chronic kidney disease. Kidney int Suppl 3:1-150.Doi:10.1038/kisup.2012.76.[Google Scholar].

[22] Patel HD, Livsey SA, Swann RA, Bukhari SS, Can urine dipstick testing for urinary tract infection at point of care reduce laboratory workload?. JClinPathol.2005;58(9):951-4. 10.1136/jcp.2004.025429 PMC1770822 16126876

[23] Schwartz GJ, Haycock GB, Edelmann CM, Spitzer A (1976). A simple estimate of glomerular filtration rate in children derived from body length and plasma creatinine. Pediatrics.

[24] Schwartz GJ, Munoz A, Schneider MF, Mak RH, Kaskel F, Warady BA, Furth SL (2009). New equations to estimate GFR in children with CKD. J Am Soc Nephrol.

[25] Colley DG,Binder S, Campbell C, King CH, Tchuem Tchuenté LA, N'Goran EK, Erko B, Karanja DMS, Kabatereine NB, van Lieshout L, Rathbun S. A five-country evaluation of a point-of-care circulating cathodic antigen urine assay for the prevalence of Schistosoma mansoni. J Trop Med Hyg . 2013 Mar; 88(3): 426-432. doi; 10.4269/ajtmh. 12-0639.Epub 2013 Jan 21. 10.4269/ajtmh.12-0639 PMC3592520 23339198

[26] Fuss A, Mazigo HD, Tappe D, Kasang C, Mueller A (2018). Comparison of sensitivity and specificity of three diagnostic tests to detect Schistosoma mansoni infections in school children in Mwanza region. Tanzania. PLoS One.

[27] Gray DJ, Ross AG, Li YS, McManus DP (2011). Diagnosis and management of schistosomiasis. BMJ.

[28] Kaze , A.D., Ilori, T., Jaar, B.G. et al. Burden of chronic kidney disease on the African continent: a systematic review and meta-analysis. BMC Nephrol 19, 125(2018) doi;10.1186/s12882-018-0930-5. 10.1186/s12882-018-0930-5 PMC5984759 29859046

[29] Kimaro FD, Jumanne S, Sindato EM, Kayange N, Chami N (2019). Prevalence and factors associated with renal dysfunction among children with sickle cell disease attending the sickle cell disease clinic at a tertiary hospital in Northwestern Tanzania. PLoS One.

[30] Jones CA, Francis ME, Eberhardt MS, Chavers B, Coresh J, Engelgau M, Kusek JW, Byrd-Holt D, Narayan KM, Herman WH (2002). Microalbuminuria in the US population: third National Health and Nutrition Examination Survey. Am J Kidney Dis.

[31] Cho H, Kim JH (2017). Prevalence of microalbuminuria and its associated cardiometabolic risk factors in Korean youth: data from the Korea National Health and Nutrition Examination Survey. PLoS One.

[32] Orlandi PF, Fujii N, Roy J, Chen HY, Lee Hamm L, Sondheimer JH, He J, Fischer MJ, Rincon-Choles H, Krishnan G (2018). Hematuria as a risk factor for progression of chronic kidney disease and death: findings from the Chronic Renal Insufficiency Cohort (CRIC) Study. BMC Nephrol.

[33] Yuste C, Gutierrez E, Sevillano AM, Rubio-Navarro A, Amaro-Villalobos JM, Ortiz A, Egido J, Praga M, Moreno JA (2015). Pathogenesis of glomerular haematuria. World J Nephrol.

[34] MugonoM, Konje E, Kuhn S, Mpogoro FJ, Morona D, Mazigo HD: Intestinal schistosomiasis and geohelminths of Ukara Island, North-Western Tanzania: prevalence, intensity of infection and associated risk factors among school children. Parasit Vectors 2014, 7:612. 10.1186/s13071-014-0612-5 PMC4297386 25533267

[35] Knopp S, Ame SM, Hattendorf J, Ali SM, Khamis IS, Bakar F, Khamis MA, Person B, Kabole F, Rollinson D (2018). Urogenital schistosomiasis elimination in Zanzibar: accuracy of urine filtration and haematuria reagent strips for diagnosing light intensity Schistosoma haematobium infections. Parasit Vectors.

